# Torque Teno Virus Viremia in Complicated *Staphylococcus aureus* Infection: Prevalence, Viral Load, and Associations With Inflammatory Markers and Clinical Outcomes

**DOI:** 10.1002/jmv.71182

**Published:** 2026-10-17

**Authors:** Fabian Ritter, Stephan Moser, Karoline Leuzinger, Michael Osthoff

**Affiliations:** ^1^ Division of Internal Medicine University Hospital Basel Basel Switzerland; ^2^ Division of Internal Medicine Inselspital, University Hospital Bern Bern Switzerland; ^3^ Department of Clinical Research University of Basel Basel Switzerland; ^4^ Clinical Virology University Hospital Basel Basel Switzerland; ^5^ Division of Internal Medicine Kantonsspital Winterthur Winterthur Switzerland

## Abstract

Torque teno virus (TTV) is a recognized marker of immune competence, but its relevance in severe bacterial infections is unclear. We evaluated prevalence, viral load, and clinical relevance of TTV viremia in complicated *Staphylococcus aureus* infection (CSAI). In this ancillary analysis of two prospective pharmacokinetic cohort studies at a Swiss university hospital, stored plasma samples were tested for TTV DNA using TTV R‐GENE PCR, and cytokines were measured by multiplex immunoassay. Associations between TTV load, inflammatory biomarkers, and clinical outcomes (including 30‐day mortality) were assessed. Among 103 patients (median age 67.9 years; 85% with bloodstream infection), TTV viremia was detected in 76% at baseline with a median viral load of 2.85 log_10_ copies/mL (IQR 1.87–3.56). Levels remained stable over 1 week despite declining inflammatory markers. Baseline TTV load was numerically higher in 30‐day non‐survivors (3.53 vs. 2.83 log_10_ copies/mL; *p* = 0.054) and not associated with mortality after adjusting for age, sex, and SOFA score (OR 1.29 per log_10_ increase; 95% CI 0.82–2.02; *p* = 0.268). TTV load was not associated with ICU admission, bacteremia duration, or length of stay. TTV viremia is common in CSAI but shows no significant association with inflammation or short‐term outcomes, suggesting limited value for risk stratification.

## 1 Introduction

Torque teno virus (TTV) is a small, non‐enveloped, single‐stranded DNA virus belonging to the *Anelloviridae* family [1]. TTV is currently considered a non‐pathogenic component of the human virome [2, 3] with prevalence rates of TTV viremia among healthy blood donors ranging from 65% to 95% [4, 5, 6].

In recent years, TTV has gained attention due to its potential to serve as a surrogate marker for the host's immune competence in solid organ transplant recipients. Several studies have shown that TTV viral load correlates with the degree of immunosuppression and may reflect the overall activity of the host's immune system. Low TTV levels early post‐transplant indicate an immune system capable of a strong response and are associated with allograft rejection (insufficient immunosuppression), whereas high TTV levels suggest a weakened immune system and are linked to an increased risk of infectious complications (excessive immunosuppression) [1, 7, 8]. Notably, an EU‐funded Horizon 2020 multicenter randomized controlled trial— TTV GUIDE IT—recently reported first results demonstrating the safety and preliminary efficacy of TTV‐guided dosing of immunosuppressive drugs in kidney transplant recipients [9, 10]. In addition, TTV DNA load has been associated with immune system activity in settings other than transplantation: it has been suggested as a predictor of immune reconstitution in HIV‐infected individuals [11], to predict response to vaccination [12], and may also be useful in predicting the occurrence of infections and mortality in COVID‐19 patients [13].

However, studies examining TTV viral load in patients with severe bacterial infections remain limited. Specifically, the presence of TTV viremia has not yet been investigated nor correlated with complications or outcome in patients with complicated *Staphylococcus aureus* infections (CSAI). CSAIs such as septic arthritis, spondylodiscitis, and endocarditis are an important cause of morbidity and mortality worldwide [14]. In particular, *S. aureus* bloodstream infection (SAB) remains a devastating disease with high mortality despite appropriate antibiotic therapy and aggressive management, including identification of infection foci, early surgery, and consultation of an infectious disease specialist [15, 16]. Host immune response plays a key role in determining clinical outcomes, and variations in immune responses may contribute to the heterogeneity of clinical outcomes in patients with SAB. Multiple studies have identified candidate proteins associated with mortality and persistent bacteremia. Nevertheless, there is still a gap in identifying reliable biomarkers for predicting clinical outcomes or treatment failure in CSAI patients.

Circulating TTV DNA in the blood may aid in the management of CSAI by contributing to risk stratification and providing information about the status of the immune system.

The primary aim of this study was to determine the prevalence of TTV viremia in patients with CSAI and describe TTV plasma loads during acute bacterial infection over 1 week. Additionally, we explored correlations between TTV viral load and molecular markers of immune activation and evaluated its association with clinical outcomes, including ICU admission, duration of bacteremia, length of stay, and 30‐day mortality.

## 2 Methods

### 2.1 Patients and Plasma Sampling

This study was conducted as an ancillary investigation using data from two single‐center, prospective, observational pharmacokinetic cohort studies carried out at a 770‐bed tertiary care hospital in Switzerland between January 2018 and December 2021. These studies aimed to assess whether contemporary dosing of the β‐lactam antibiotic flucloxacillin or cefazolin achieves pharmacological target attainment in patients with CSAI caused by methicillin‐susceptible *S. aureus* (MSSA).

Patients with CSAI were included if aged >18 years and treated with flucloxacillin or cefazolin during hospitalization. Exclusion criteria were polymicrobial infection, hemodialysis treatment, and pregnancy. CSAI was defined as MSSA bloodstream infection (i.e., at least one MSSA‐positive blood culture), the presence of an infection site remote from the primary focus caused by hematogenous seeding (e.g., endocarditis, vertebral osteomyelitis), extension of the infection beyond the primary focus (e.g., septic thrombophlebitis or abscess), or deep‐seated infections caused by MSSA (e.g., osteoarticular infections, deep‐seated abscesses). Detailed inclusion/exclusion criteria have been previously published [17, 18].

For this study, we retrospectively selected all patients from the two cohorts for which at least one stored EDTA plasma sample was available for quantitative analysis of TTV DNA and cytokine concentrations. Both principal studies included blood sampling at several time points during hospital admission, including study days 1, 3, and 7 (Supporting Information S1: Figure S1). Samples were centrifuged, aliquoted, and stored at −80°C immediately after blood collection. Both principal studies were approved by the Ethics Committee of Northwest and Central Switzerland (EKNZ Project‐ID: 2017‐02072 and EKNZ Project‐ID: 2019‐02229). All patients provided written informed consent for inclusion in this sub‐study.

Demographic, clinical, and laboratory data were prospectively collected and recorded in a secure database.

### 2.2 TTV DNA and Cytokines Quantitation

TTV DNA was extracted from plasma samples using the MagNaPure 96 (Roche Diagnostics, Rotkreuz, Switzerland). DNA amplification and quantification were performed using the Argene TTV R‐GENE assay kits (BioMerieux, France) on an Applied Biosystem 7500 (Thermofisher, Waltham, MA), following the manufacturer's instructions. The threshold for defining positivity was a CT value < 40. Results are expressed as log_10_ copies/mL. For analysis purposes, specimens with undetectable or negative TTV DNA (i.e., CT value > 40) were assigned a value of 1 log_10_ copies/mL.

Plasma cytokine concentrations and markers of the host immune system (TNF‐α, IL‐1β, MCP‐1, IL‐6, IL‐8, IL‐10, IL‐17A) were quantified by using a high‐sensitivity, customized multiplex assay on the Meso Scale Discovery (MSD) U‐PLEX platform, as per the manufacturer's standard protocol. All plasma samples were assayed in duplicates for TTV viral load and cytokine quantification.

### 2.3 Statistical Analysis

Descriptive analysis was performed for all variables. Categorical data are presented as frequencies with percentages or continuous variables as median with interquartile ranges (25th–75th percentile). Frequency comparisons for categorical variables were carried out using *χ* ^2^ test or Fisher's exact test, as appropriate. Differences between medians were assessed using the Mann–Whitney U test or the Wilcoxon signed‐rank test, as appropriate. Correlations between variables of interest were evaluated using Spearman's Rank test. To evaluate the association between baseline TTV viral load and 30‐day mortality, binary logistic regression analyses were performed using baseline TTV viral load as a continuous variable. Univariable and multivariable models were fitted, with the latter additionally adjusting for age, sex, and SOFA score at CSAI onset. Odds ratios (ORs) with 95% CI were calculated.

Statistical significance was defined as a two‐sided *p* value < 0.05. All data were analyzed using SPSS, version 28.0 (IBM; Armonk, NY, USA).

### 2.4 Declaration of Generative AI Use

During the preparation of this work, the authors used Chat GPT‐5 mini as well as OpenEvidence in order to generate ideas to discuss and improve language and readability. The authors reviewed and edited the AI‐generated output and take full responsibility for the content of the published article. We ensure the manuscript represents the author's authentic and original contribution and reflects our own analysis, interpretation and insights.

## 3 Results

### 3.1 Cohort

Between January 2018 and December 2021, a total of 103 CSAI patients (28 women and 75 men) were included in this sub‐study, with a median age of 67.9 years. The median time from the collection of the first positive bacterial culture to patient inclusion (Day 1) was 4 days (IQR 3–6). At enrollment, patients had received antibiotic treatment for a median of 4 days (IQR 3–6). Eighty‐eight patients (85%) had MSSA bloodstream infections. Baseline characteristics are summarized in Table 1.

**Table 1:** Characteristics and outcome of 103 patients with CSAI.

		*n* (%) or median (IQR)	
Age (years)		67.9 (53.8–79.8)	
Female sex		28 (27)	
BMI (kg/m^2^)		25.5 (21.7–29.2)	
Length of stay (days)		22 (16–39)	
Comorbidities			
Diabetes mellitus		23 (22)	
Liver cirrhosis		7 (7)	
Chronic kidney disease		23 (22)	
Cardiovascular disease		44 (43)	
Chronic lung disease		16 (16)	
Malignancies		12 (12)	
IV Drug use		14 (14)	
Immunosuppression		5 (5)	
Charlson comorbidity score		4 (2–6)	
Disease severity	At CSAI onset		At study inclusion
Pitt bacteraemia score	0 (0–1)		0 (0–1)
SOFA score	2 (1–4)		2 (0–4)
Laboratory results			
C‐reactive protein (mg/L)	153 (46–248)		107 (43–170)
Leukocytes (10^6^/L)	12.5 (9.1–16.5)		8.9 (6.6–12.4)
Creatinine (µmol/L)	81 (68–137)		73 (62–105)
eGFR (mL/min/1.73 m^2^)	76 (38–96)		87 (55–103)
Focus of CSAI			
Skin and soft tissue		26 (25)	
Endocarditis		24 (23)	
Catheter or foreign material		19 (18)	
Osteomyelitis or arthritis		17 (17)	
Respiratory tract		4 (4)	
Intra‐abdominal		1 (1)	
Urinary tract		1 (1)	
Other/unknown		11 (11)	
Severity and outcome			
Bloodstream infection		88 (85)	
Prolonged bacteraemia > 3 days		17 (17)	
ICU admission		32 (31)	
Vasoactive treatment		7 (7)	
30‐Day mortality		12 (12)	

*Note:* Data are number of patients (%) or median (IQR).

Abbreviations: BMI, body mass index; CSAI, complicated *Staphylococcus aureus* infections; eGFR, estimated glomerular filtration rate; ICU, intensive care unit; IV, intravenous; IQR, interquartile range; SOFA, sequential organ failure assessment.

Skin and soft tissue infections were the most common sources of CSAI (*n* = 26; 25%), followed by infective endocarditis (*n* = 24; 23%), infections related to catheters or foreign material (*n* = 19; 18%), and bone or joint infections (*n* = 17; 17%). The median length of hospital stay was 22 days (IQR 16–39). Twelve patients (12%) died within 30 days after CSAI onset.

### 3.2 Prevalence of TTV Viral Load, and Its Relative Short‐Term Stability

At study inclusion, TTV DNA was detected in plasma from 78 patients (76%), with a median baseline viral load of 2.85 log_10_ copies/mL (IQR 1.87–3.56). A higher proportion of women were TTV‐negative compared to men (43% vs. 16%; *p*= 0.008). During the following 7 days, two additional patients became TTV positive. Overall, 78% of patients had at least one detectable episode of TTV viremia. Median TTV DNA load and levels across study days 1, 3, and 7 are depicted in Figure 1.

**Figure 1:**
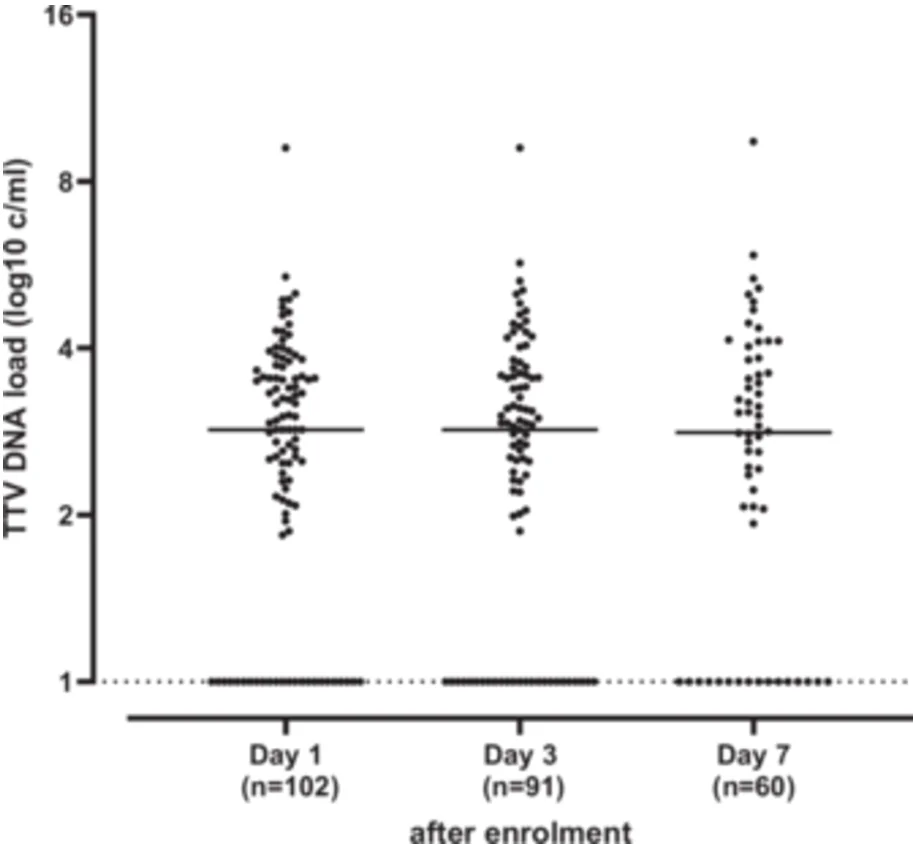
Comparison of the TTV DNA load according to the study day. Horizontal lines represent the median of the TTV DNA load in log_10_. Dashed line: limit of detection.

TTV plasma load in CSAI patients remained stable over the course of 1 week with only minimal variation in intra‐individual levels. In contrast, significant inter‐individual differences were observed. The highest TTV DNA load was measured in an immunosuppressed patient receiving maintenance therapy with rituximab for mantle cell lymphoma (9.44 log_10_ copies/mL). No significant differences in baseline characteristics were observed between patients with detectable and undetectable viral load, except for sex distribution (Supporting Information S1: Table S1).

### 3.3 Association of TTV Viral Load With Clinical Outcomes

Baseline median TTV plasma load (Day 1) was numerically higher in patients who died (median: 3.53 log_10_ copies/mL; IQR 2.85–4.42) compared with survivors (median: 2.83 log_10_ copies/mL; IQR 1–3.54), with a Hodges–Lehmann median difference of 0.00 to 1.68 log_10_ copies/mL (95% CI; *p*= 0.054, Figure 2).

**Figure 2:**
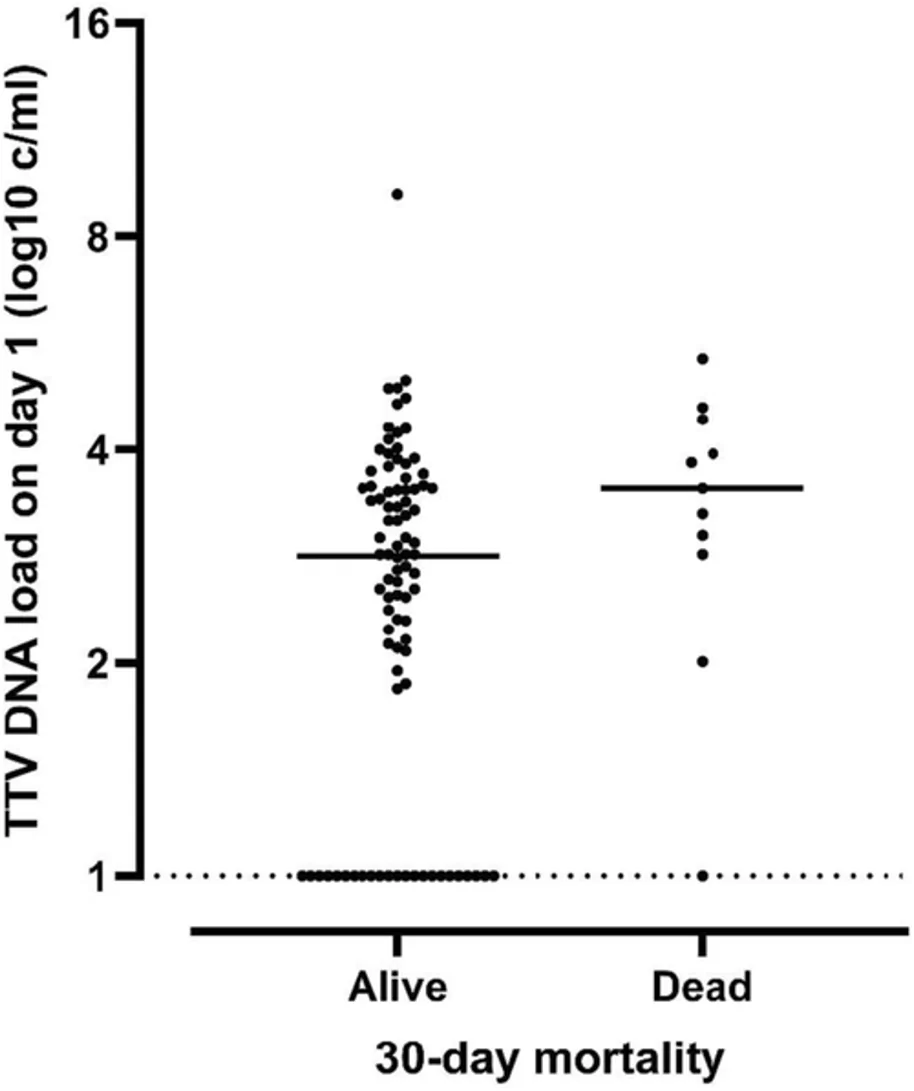
TTV DNA viral load on Day 1 according to 30‐day mortality after diagnosis of CSAI.

To evaluate the potential influence of immunosuppression on our findings, we performed a subgroup analysis excluding immunosuppressed patients (*n* = 5), which yielded similar results (*p* = 0.063).

In univariable logistic regression, each 1 log_10_ increase in baseline TTV viral load was associated with an estimated odds ratio of 1.41 for 30‐day mortality (95% CI 0.92–2.15; *p* = 0.115). In the multivariable logistic regression model adjusting for age, sex, and SOFA score at CSAI onset, higher baseline TTV viral load was associated with increased odds of 30‐day mortality but did not reach statistical significance (OR 1.29 per 1 log_10_ increase; 95% CI 0.82–2.02; *p* = 0.268). Age was the only variable significantly associated with 30‐day mortality in the multivariable model (OR 1.08 per year; 95% CI 1.01–1.16; *p* = 0.017).

Baseline TTV viral load was not significantly associated with ICU admission, length of hospital stay, or duration of bacteraemia.

### 3.4 Pro‐/Anti‐Inflammatory Biomarkers and TTV DNA Load

Median levels of proinflammatory biomarkers (CRP, IL‐1β, IL‐6, and IL‐17A) declined gradually during hospitalization, consistent with resolution of acute inflammation (Supporting Information S1: Figure S2), while no clear trends were apparent for IL‐8, IL‐10, TNF‐α, and MCP‐1 (data not shown). TTV DNA did not correlate with plasma levels of any of these biomarkers except for a weak negative correlation with IL‐1β. Likewise, TTV viral load showed no relevant relation with disease severity as reflected by the SOFA score at CSAI onset (Figure 3).

**Figure 3:**
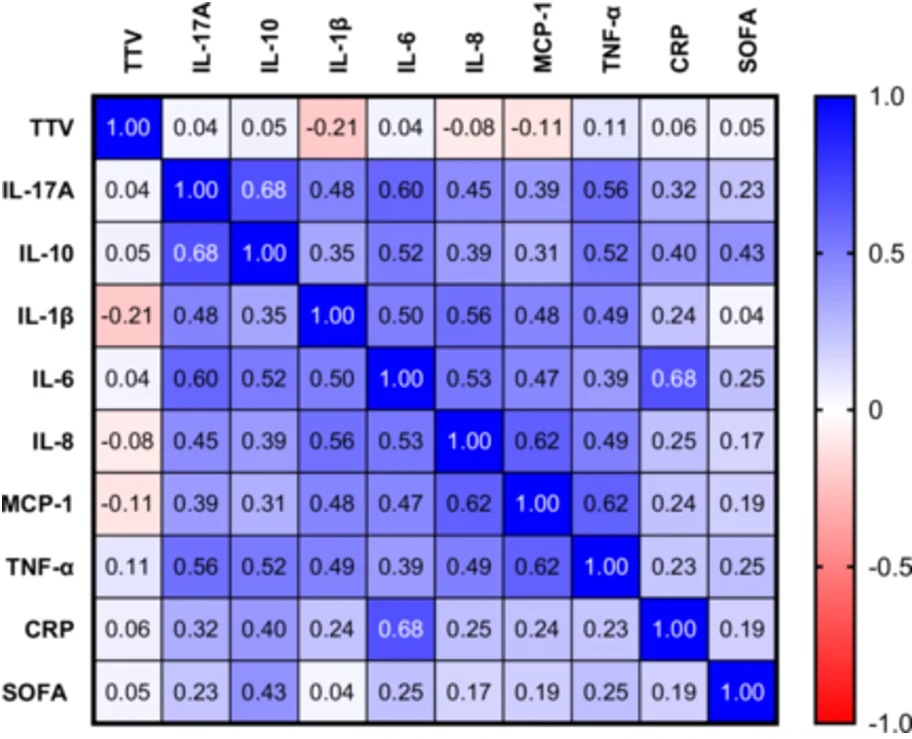
Correlation coefficients in CSAI patients on first study visit. CRP, C‐reactive protein; SOFA, sequential organ failure assessment score.

## 4 Discussion

In this exploratory sub‐study of patients with complicated *Staphylococcus aureus* infections, we investigated the prevalence, viral load, and clinical relevance of plasma TTV DNA. Our data provide several important insights. First, TTV viremia was highly prevalent in this cohort, affecting more than three‐quarters of patients. Second, TTV plasma viral loads remained remarkably stable over a 1‐week hospitalization course despite pronounced changes in inflammatory biomarkers. Third, TTV DNA load showed no significant association with disease severity, inflammatory markers, or most clinical outcomes, despite a non‐significant trend toward higher baseline TTV levels in non‐survivors.

The high prevalence of TTV viremia observed in our cohort (78%) is consistent with previous reports demonstrating widespread TTV detection across diverse patient populations and healthy individuals, highlighting the ubiquitous nature of this virus [4, 5].

Interestingly, we observed a significantly higher proportion of TTV‐negative individuals among women compared to men. Similar gender differences have been reported previously and may reflect biological and immunological factors [4, 19, 20]. For instance, hormonal fluctuations have been implicated in modulating TTV susceptibility. One study found that lower progesterone levels in anovulatory women were associated with increased TTV detection, suggesting that sex hormones may influence viral replication and persistence [19]. However, in the present cohort, median viral loads did not differ significantly between TTV‐positive men and women. Nevertheless, our data suggest that biological sex may represent a modifier of TTV persistence and warrants further investigation.

TTV plasma loads remained stable over the 1‐week observation period, with only minimal intra‐individual variability. In our cohort, neither baseline nor TTV measurements over a course of 7 days were associated with a broad panel of inflammatory markers, despite the expected decline of these biomarkers during clinical recovery. Previous studies investigating associations between TTV load and inflammatory biomarkers have yielded heterogeneous results. While recent work in viral respiratory infections reported selected age‐dependent correlations between TTV and cytokines such as IL‐6 and TNF‐α, these findings were inconsistent across patient subgroups, and their biological significance remains unclear [21]. In line with observations from critically ill COVID‐19 cohorts, we did not identify meaningful associations between TTV levels and a broad panel of inflammatory biomarkers in CSAI [13].

The stability of TTV levels in our cohort suggests that acute bacterial infection and antibiotic exposure do not immediately alter the host mechanisms controlling TTV replication. Alternatively, the 1‐week observation period may not have been sufficient to capture potential changes occurring over a longer time frame. Consistent with observations in transplant recipients, TTV viral loads do not appear to change substantially during the first days following the initiation or adaptation of immunosuppressive therapy, with significant increases becoming evident only after several weeks [22, 23]. However, short‐term alterations may be biologically plausible in the setting of acute infection as TTV replication has been linked to immune cell populations, including neutrophilic granulocytes [24, 25]. In our cohort, despite the profound inflammatory response associated with CSAI, we did not observe relevant changes in TTV levels over the 1‐week observation period, suggesting that TTV monitoring may have limited utility for short‐term assessment of immunocompetence during acute bacterial infection. Future studies with longer observation periods are needed to determine whether TTV levels change over more extended time frames in a severe condition such as CSAI.

Regarding clinical outcomes, we observed a non‐significant trend toward higher baseline TTV viral loads in patients who died within 30 days. In multivariable logistic regression adjusted for age, sex, and SOFA score at CSAI onset, the direction of this association was unchanged from the univariable mode, although not reaching statistical significance. Our findings, therefore, do not establish baseline TTV viral load as an independent predictor of mortality but support the hypothesis that higher TTV levels may reflect impaired immune competence.

Adjustment for age and sex is particularly relevant in this context, as both are recognized determinants of TTV replication in the general population. In a large cohort of healthy blood donors, TTV prevalence rose steadily with age and was higher in men than in women [4]. Another cohort of healthy adults found that plasma TTV load increased significantly with age, with the lowest levels in young women [5]. Thus, differences in age and sex may contribute to variation in TTV levels and should be accounted for when assessing the potential role of TTV in risk stratification.

Our findings are also broadly consistent with previous reports linking higher TTV levels to adverse outcomes in critically ill and elderly populations [13, 26, 27]. In critically ill patients with COVID‐19, detectable TTV DNA was associated with an increased risk of subsequent infectious events, while higher TTV levels showed a non‐significant trend toward increased mortality [13]. Moreover, a recent study in immunocompromised patients admitted to the ICU for acute respiratory failure demonstrated that higher TTV burden in nasopharyngeal swabs was associated with increased rates of pulmonary infections and higher mortality [26]. Taken together, these findings support the potential relevance of TTV as a marker of impaired immune competence across different high‐risk populations, while differences in patient characteristics, sampling compartments, and clinical outcomes warrant cautious interpretation of cross‐study comparisons.

Our findings should nevertheless be interpreted cautiously. Non‐survivors were older and had greater disease severity, and the number of fatal events was small (Supporting Information S1: Table S2). Notably, age was the only variable significantly associated with 30‐day mortality in the multivariable model (OR 1.08 per year, 95% CI 1.01–1.16; *p* = 0.017). Furthermore, baseline TTV levels were not significantly associated with other clinical outcomes, including ICU admission, length of hospital stay, or duration of bacteremia.

Our study has several limitations. First, the single‐center design limits external validity. Second, the overall sample size was modest, and the analysis of 30‐day mortality is limited by the small number of fatal events (*n* = 12), which reduced statistical power and precision. Although baseline TTV loads were numerically higher in non‐survivors, this observation should be interpreted with caution and does not establish an added role for baseline TTV viral load in risk stratification. Third, the predominance of male patients in the cohort may further restrict the generalizability of our findings. Fourth, study inclusion occurred at a median of 4 days after the first positive bacterial culture, and patients had already received several days of antibiotic therapy at baseline. Therefore, early immunological dynamics at infection onset may not have been captured. Finally, the observation period was limited to a 1‐week period and may have been too short to capture delayed effects on TTV replication.

## 5 Conclusion

In this cohort of patients with complicated *Staphylococcus aureus* infections, TTV viremia was highly prevalent, with TTV viral loads remaining stable during a 1‐week hospitalization period. Despite its proposed role as a surrogate marker of immune competence in other clinical settings, TTV viral load was not significantly associated with inflammatory biomarkers, disease severity, or most short‐term clinical outcomes. Overall, our findings suggest limited added value of TTV load for risk stratification beyond established clinical parameters in acute complicated *S. aureus* infections, while we await data from larger studies with a longer period of TTV viral load measurement.

## Ethics Statement

Both principal studies were approved by the Ethics Committee of Northwest and Central Switzerland (EKNZ Project‐ID: 2017‐02072 and EKNZ Project‐ID: 2019‐02229).

## Supporting information


Supporting File

## Data Availability

Data are available from the corresponding author upon reasonable request.
