# Supplementary material for: Torque Teno Virus Viremia in Complicated Staphylococcus aureus Infection: Prevalence, Viral Load, and Associations With Inflammatory Markers and Clinical Outcomes
Source: J Med Virol. 2026 Oct 17;98(10):e71182. doi: 10.1002/jmv.71182 (PMC13675932; doi:10.1002/jmv.71182)
Supplement: Supplementary file 1 [file JMV-98-e71182-s001.docx]

**Supplementary data**

**Supplemental Fig. 1:** Flow chart of available plasma samples for TTV measurement

**Supplemental Fig. 2:** Course of C-reactive protein and IL-6 (A), and IL-17A and IL-1β (B) during the study period. Medians and interquartile ranges are depicted.

| Supplemental Table 1 Comparison of patient characteristics stratified according to TTV DNA detection at study enrolment | | | | |
| --- | --- | --- | --- | --- |
|  | **Yes** (n=78) ^†^ | **No**  (n=24) |  | *p value** |
| Age in years, median (IQR) | 68.0 (53.5–80.1) | 67.2 (55.5–79.3) |  | 0.78 |
| Female sex | 16 (21) | 12 (50) |  | 0.008^#^ |
| Charlson comorbidity score | 4 (2–7) | 4 (1–6) |  | 0.36 |
| Serum creatinine (µmol/l) | 73 (61–115) | 77 (62–102) |  | 0.91 |
| eGFR (mL/min/1.73 m^2^) | 87 (54–103) | 82 (54–93) |  | 0.68 |
| SOFA score | 2 (0–4) | 2 (1–3) |  | 0.92 |
| C-reactive protein (mg/L) | 105 (52–170) | 89 (32–165) |  | 0.29 |
| IL-6 (pg/mL) | 13.0 (4.6–32.8) | 9.1 (6.3–18.4) |  | 0.40 |
| IL-1β (pg/mL) | 0.27 (0.15–0.57) | 0.50 (0.18–0.63) |  | 0.14 |
| IL-10 (pg/mL) | 0.98 (0.49–2.32) | 0.91 (0.57–1.76) |  | 0.93 |
| Length of stay (days) | 22 (16–39) | 20 (14–38) |  | 0.76 |

^†^one sample was missing for baseline TTV measurement; *Mann-Whitney-U-Test; ^#^Fisher’s-exact test

Data are number of patients (%) or median (IQR). IQR, interquartile range; SOFA, sequential organ failure assessment; eGFR, estimated glomerular filtration rate

| Supplemental Table 2 Comparison of patient characteristics stratified according to survival outcome | | | | |
| --- | --- | --- | --- | --- |
|  | **Survived** (n=91) | **Died**  (n=12) |  | *p value* |
| Age in years, median (IQR) | 64.2 (53.3–79.8) | 78.2 (75.4–84.3) |  | 0.013* |
| Female sex | 25 (27) | 2 (17) |  |  |
| Disease severity at study inclusion |  |  |  |  |
| Pitt bacteraemia score | 0 (0–1) | 2 (0–5) |  | <0.001* |
| SOFA score | 2 (0–3) | 6 (2–10) |  | <0.001* |
| Laboratory results |  |  |  |  |
| C-reactive protein (mg/L) | 99 (41–164) | 168 (115–217) |  | 0.06 |
| leukocytes (10^6^/L) | 8.8 (6.5–12.5) | 9.6 (8.6–12.1) |  | 0.33 |
| TTV load and immunologic biomarkers on study day 1 |  |  |  |  |
| TTV DNA load (log_10_ copies/mL) | 2.83 (1–3.53) | 3.53 (2.94–4.19) |  | 0.06 |
| IL-1β (pg/mL) | 0.28 (0.17–0.57) | 0.71 (0.21–0.96) |  | 0.10 |
| IL-6 (pg/mL) | 10.0 (4.2–29.0) | 40.3 (20.5–80.7) |  | 0.003* |
| IL-10 (pg/mL) | 0.86 (0.48–1.84) | 2.32 (1.04–3.83) |  | 0.02* |
| changes in TTV viral load from day 1 to day 3 (Δ TTV) ^#^ | -0.01(-0.22–0.13) | 0.13 (-0.14–0.38) |  | 0.701 |

Data are number of patients (%) or median (IQR). IQR, interquartile range; SOFA, sequential organ failure assessment;

^#^ Only patients with detectable TTV and available measurements at day 1 and 3 (survivors n=58, non-survivors n=8)
